# Editorial: Cellular processes in placental morphogenesis

**DOI:** 10.3389/fcell.2023.1298298

**Published:** 2023-10-04

**Authors:** Claudio Gustavo Barbeito, Maria Angélica Miglino

**Affiliations:** ^1^ Laboratory of Descriptive, Experimental and Comparative Histology and Embryology, School of Veterinary Sciences, National University of La Plata, National Scientific and Technical Research Council, La Plata, Argentine; ^2^ Department of Surgery, School of Veterinary Medicine and Animal Science, University of São Paulo, São Paulo, Brazil

**Keywords:** placenta, decidua, trophoblast, adaptation, mother-conceptus interface

The placenta is a temporary and mixed organ (maternal and embryonic/fetal origin), which appeared several times during the evolution of vertebrates and represents one of the most complex adaptations to matrotrophy. In fish and reptiles, it emerged repeatedly in distinct events and with very diverse features, while it originated in a single moment for mammal evolution, specifically in a common ancestor between metatheria and eutheria (Griffith, 2021). After its appearance, although the organ was present throughout the evolutionary history of these groups, the placenta diversified more than any other organ. This variability was attributed to different factors, but probably depends to a large extent to the expression of retroviral genes (Imakawa et al., 2022). One of the aspects in which placentas of different species differ is the number of layers separating maternal blood from embryonic/fetal blood. Epitheliochorial placentas have six layers (maternal endothelium, maternal connective tissue, endometrial epithelium, trophoblast, fetal mesenchyme and fetal endothelium); in endotheliochorial placentas the epithelium and connective tissue of the maternal endometrium disappear and in hemochorial placentas there are no maternal layers. Currently, it is considered that the basal placenta of eutherians was very invasive and can be classified as hemochorial. This placenta is thought to be similar to that found in present rodents, lagomorphs and many primates such as human, and already had a change in the endometrial connective tissue known as decidual reaction. Later, endotheliochorial placentas -as those of most carnivorous species, pachyderms and sirenids-arose, maintaining the maternal endothelium and epitheliochoriol placentas without the loss of layers that exists in perissodactyls, cetartiodactyls and some primates, such as lemurs (Carter, 2018). Beyond phylogenetic variation in structural aspects, placentas are also very different in terms of transport, endocrinological and immunological functions; as well as in its adaptive capacity and permeability.

Placental diversity is found both in the fetal component, especially in the trophoblast, and in the decidua, which is of maternal origin. The trophoblast has different cell types in different species; in rodents this diversity is outstanding. In this Research Topic, Favaron and Carter review the existing data on trophoblastic giant cells in cricetids, bringing together results from various authors including themselves. It is remarkable how the authors describe in detail the differences between murids and cricetids. Since, in many cases, results from mice are extrapolated to other rodents, a look into their differences, even when it comes to the sister group of murids, is a relevant contribution to the field. It is interesting to note that cricetids include species used as experimental models, such as the Hamster and the Gerbil, and that, as the authors point out, they also represent 15% of living mammal species.

Regarding the decidua, although decidualization is much more extensive in hemochorial placentas, it also exists in many of the endotheliochorial placentas specifically with the differentiation of fibroblast to decidual stromal cels (DSC). Diessler et al. note that DSC have some common markers in different carnivores, but their comparative analysis reveals that differ in some aspects between Canifornia and Feliformia. Although DSC are much more evident in felines, current data irrefutably demonstrate their existence in canines, which was under discussion until few decades ago (Fernández et al., 2000). These cells not only have roles in maintaining pregnancy, but also participate in the induction of luteolysis, which is essential for triggering parturition in these species.

Eutherian have adapted to living in different environments with conditions in which the availability of oxygen is less than the usual. Some organisms live at high altitudes where the atmospheric pressure and, consequently, the partial pressure of oxygen, are very low. Others spend a long time submerged without breathing movements. Among animals that perform long dives, sirenids and pinnipeds have an endotheliochorial placenta and cetaceans have an epitheliochorial placenta (Carter, 2018), similar to what occurs in related terrestrial species. However, some specific adaptations were found in a pinniped species that performs long apneas for diving, the southern elephant seal *Mirounga leonina*. In this species, the placenta is characterized by large maternal sinusoid capillaries and indentations of the cytotrophoblast in the maternal endothelium, adaptations that improve maternal-fetal contact (Diessler et al., 2020). Regarding phenotypic variation as a response to altitude, inter and intraspecific adaptations were studied. For example, the placenta of the alpaca (*Vicugna pacos*) has a shorter interhemal distance than other epitheliochorial cases and there are also numerous areas in which the vessels are indented in the epithelium (Olivera et al., 2003). Here, Navarrete Zamora et al. finds some variations in the glycosylation pattern of the alpaca trophoblast that would not respond to phylogenetic factors, as found in many species (Acuña et al., 2023). Regarding intraspecific variations, the human placenta of populations adapted to high altitudes for centuries has different morphological and genetic changes (Zamudio, 2003). These variants would also be found in the regulation of gene expression. In this line, Gundling et al. reports differences in the methylation of a gene that codes for dysferlin, a protein involved in syncytium formation in human populations adapted to high altitudes.

Finally, the placenta responses to various injuries. Alcohol is recognized as one of the main teratogenic agents; its effect on embryonic development, especially to the neural crest, has been studied for decades (Chen et al., 2021). More recently, it has been shown that, in addition to this direct effect on embryogenesis, alcohol causes damage to the placenta. Gualdoni et al. review this Research Topic and offer an insight into the harmful effects of ethanol on angiogenesis by analyzing the role of the metalloproteinase and nitric oxide pathways. This review is focused on the mouse and, although its placenta with three layer of trophoblast is less similar to the human one than that of hystricomorph rodents, which have a single layer of trophoblast separating the circulations (Flamini et al., 2011), the use of mouse in biomedical research is predominant and many results could be extrapolated to human.

The variability of the placenta is enormous, as its capacity to adapt and respond to injuring agents. In this Research Topic we aim to cover part of the current knowledge produced in this area.
